# DNA methylation dynamics in mouse preimplantation embryos revealed by mass spectrometry

**DOI:** 10.1038/srep19134

**Published:** 2016-01-11

**Authors:** Yoshinori Okamoto, Naoko Yoshida, Toru Suzuki, Nobuhiro Shimozawa, Maki Asami, Tomonari Matsuda, Nakao Kojima, Anthony C. F. Perry, Tatsuyuki Takada

**Affiliations:** 1 Faculty of Pharmacy, Meijo University, 150 Yagotoyama, Tempaku-ku, Nagoya 468-8503, Japan; 2 Department of Stem Cell Pathology, Kansai Medical University, 2-5-1 Shinmachi, Hirakata, Osaka 573-1010, Japan; 3 Laboratory of Mammalian Molecular Embryology, Department of Biology and Biochemistry, University of Bath, BA2 7AY, England; 4 Tsukuba Primate Research Center (TPRC), National Institute of Biomedical Innovation (NIBIO), 1-1 Hachimandai, Tsukuba, Ibaraki 305-0843, Japan; 5 Research Center for Environmental Quality Management, Kyoto University, 1-2 Yumihama, Otsu, Shiga 520-0811, Japan; 6 Laboratory of Cell Engineering, Department of Pharmaceutical Sciences, Ritsumeikan University, 1-1-1 Nojihigashi, Kusatsu, Shiga 525-8577, Japan

## Abstract

Following fertilization in mammals, paternal genomic 5-methyl-2′-deoxycytidine (5 mC) content is thought to decrease *via* oxidation to 5-hydroxymethyl-2′-deoxycytidine (5 hmC). This reciprocal model of demethylation and hydroxymethylation is inferred from indirect, non-quantitative methods. We here report direct quantification of genomic 5 mC and 5 hmC in mouse embryos by small scale liquid chromatographic tandem mass spectrometry (SMM). Profiles of absolute 5 mC levels in embryos produced by *in vitro* fertilization (IVF) and intracytoplasmic sperm injection (ICSI) were almost identical. By 10 h after fertilization, 5 mC levels had declined by ~40%, consistent with active genomic DNA demethylation. Levels of 5 mC in androgenotes (containing only a paternal genome) and parthenogenotes (containing only a maternal genome) underwent active 5 mC loss in the first 6 h, showing that both parental genomes can undergo demethylation independently. We found no evidence for net loss of 5 mC 10–48 h after fertilization, implying that any passive ‘demethylation’ following DNA replication was balanced by active 5 mC maintenance methylation. However, levels of 5 mC declined during development after 48 h, to 1% (measured as a fraction of G-residues) in blastocysts (~96 h). 5 hmC levels were consistently low (<0.2% of G-residues) throughout development in normal diploid embryos. This work directly quantifies the dynamics of global genomic DNA modification in mouse preimplantation embryos, suggesting that SMM will be applicable to other biomedical situations with limiting sample sizes.

The mammalian genome contains 5-methyl-2′-deoxycytidine (5 mC) produced by the methylation of 2′-deoxycytidine (C), typically in the dinucleotide, CpG^1^. Genomic 5 mC provides a tier of transcriptional regulation that contributes to cell- and tissue-specific expression; as an approximation, promoters with higher 5 mC levels are less active^2^,^3^.

In the mouse, *de novo* DNA methylation is catalyzed by DNA methyl-transferase 3a (Dnmt3a) and Dnmt3b and maintained by Dnmt1 (refs 4 and 5). The dynamics of DNA methylation are important because they critically influence interactions between DNA and gene regulatory proteins^6^. Genomic 5 mC is recognized by methyl-DNA binding proteins that recruit histone deacetylases^7^, and Dnmt1, Dnmt3a and Dnmt3b respectively interact with H3 lysine 9 methyl transferases, G9a, Suv39h1 and Eset^8^,^9^,^10^. Trimethyl-H3K9 (H3K9me3) is bound by heterochromatin 1 (HP1) which recruits Dnmt1 (refs 9 and 11). Since promoters bound by deacetylated histones, H3K9me2 and H3K9me3 tend to be inactive, these histone modifications dynamically couple 5 mC to transcriptional repression. Contrastingly, unmethylated DNA is associated with acetylated histones, which facilitate transcription^12^. Unmethylated CpG dinucleotides are bound by CXXC finger protein 1 (CFP1) which recruits the H3K4 methyl-transferase Setd1 (ref. 13); H3K4me2 and H3K4me3 are also associated with transcriptional activation. Setd1-mediated H3K4 methylation is linked to the removal of silencing marks H3K9me1, H3K9me2, H3K27me2 and H4K20me1 *via* Phf8 (ref. 14).

Immunofluorescence microscopy using antibodies against 5 mC indicates that soon after fertilization, 5 mC decreases markedly in the sperm-derived genome independently of DNA replication or pronucleus formation^15^,^16^,^17^,^18^,^19^,^20^,^21^,^22^,^23^. The maternal factor, Stella (also known as Pgc7 or Dppa3) protects the maternal genome from demethylation, and also protects differentially methylated regions (DMRs) of some imprinted and non-imprinted genes in both parental genomes^21^,^24^. Recently, bisulfite sequencing has suggested that the maternal genome also undergoes active zygotic 5 mC depletion^18^,^22^. Passive depletion of 5 mC in both parental genomes occurs from the first zygotic S-phase until the blastocyst stage *via* its dilution by semi-conservative DNA replication^25^,^26^. Levels of 5-hydroxymethyl-2′-deoxycytidine (5 hmC) in paternal zygotic genomes putatively increase as 5 mC levels decrease *via* Ten-eleven translocation 3- (Tet3-) mediated oxidation^19^,^23^, decreasing in subsequent preimplantation development^27^.

Comparison between 5 mC and 5 hmC levels will reveal whether their regulation is linked, but immunofluorescence microscopy, which was initially applied to visualize zygotic 5 mC and 5 hmC, is in general suboptimal for quantification^28^. Moreover, the pronounced pre-S-phase reduction in 5 mC suggested by immunofluorescence microscopy^15^ sometimes but not always correlates with bisulfite sequencing analyses of multi-copy genomic elements including LINEs and early retroposons^29^,^30^,^31^. In addition, 5 mC and 5 hmC are indistinguishable by standard bisulfite sequence analysis^32^.

No direct quantitative method has been applied to measure 5 mC or 5 hmC content in preimplantation embryos and the extent to which zygotic 5 mC is depleted remains unclear. To date, tandem spectrometric (ms/ms) methods to quantify 5 mC and 5 hmC have not been evaluated for small sample sizes such as those available for the study of mouse preimplantation development^33^,^34^. This is because conventional ms/ms requires >20 fmol for accurate quantification of 5 mC and 5 hmC; each mature, metaphase II (mII) oocyte contains 200 and 8 amol of each respectively.

To address this, we have developed a sensitive, high-throughput small-scale liquid chromatography (lc) ms/ms (SMM) method for 5 mC and 5 hmC quantification and apply it to provide an accurate description DNA methylation in early mouse embryogenesis. Definitive 5 mC and 5 hmC quantification by SMM produces early developmental profiles that are partly compatible with data obtained by conventional methods, but are incompatible with the reciprocal model of oxidative 5 mC removal that they suggest for biparental diploid embryos.

## Results

### Standardization of SMM for 5 mC and 5 hmC

**Figure 1:**
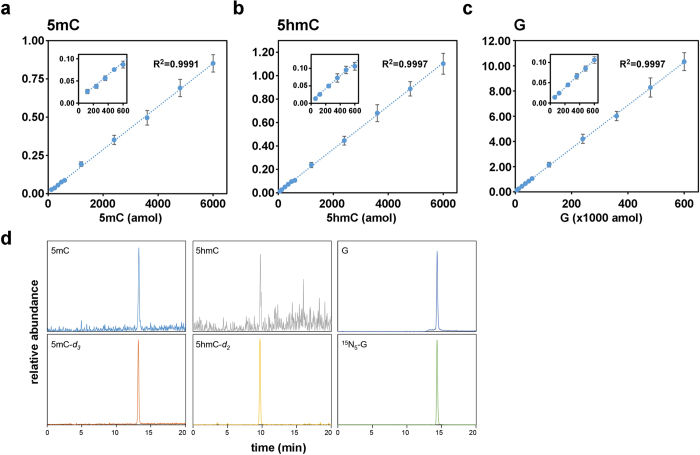
Standard curves of 2′-deoxyguanosine (G), 5-methyl-2′deoxycytidine (5 mC) and 5-hydroxymethyl-2′-deoxycytidine (5 hmC). Standard curves for (**a**) 5 mC, (**b**) 5 hmC and (**c**) G and (**d**) representative SMM chromatograms. Specific monitoring of target nucleosides was accomplished based on nucleoside-specific fragmentation, which is the loss of deoxyribose-moiety (*m/z* 116) from each parent ion. Peak area was normalized by dividing net peak area of each target by that of its internal standard. X-axes in (**a–c**) represent the quantity (amol) of injected nucleoside and Y-axes plot the ratio of each nucleoside to the labelled standard.

Quantification of DNA methylation in preimplantation embryos must be highly sensitive since the sample sizes are so small (typically ≤100 cells). We therefore adapted ms/ms for ultrasensitive quantification of 5 mC and 5 hmC by modifying sample preparation and chromatography for analysis with a high-sensitivity detection system (see Methods). This method, small-scale ms/ms (SMM), yielded detection thresholds of 6 fmol (deoxyguanosine, G), 120 amol (5 mC) and 60 amol (5 hmC) (Fig. 1a–c) and values for 5 mC and 5 hmC content in different tissues that were similar to previous estimates (Supplementary Fig. S1)^35^,^36^. To estimate its sensitivity, we applied SMM to 10 ~ 1000 mouse ES cells and obtained stable measurements for 5 mC of 3.54% (of genomic G) from 100 cells, and 0.33% for 5 hmC in 100 cells (Supplementary Fig. S2). A value for 5 mC of 3.5% agrees with an estimate for mouse ES cells grown in similar (non-2i) conditions^37^. Validation of SMM for quantification of DNA modifications in ≤100 diploid cells paved the way for its application to mouse preimplantation development.

### Direct measurement of 5 mC during mouse preimplantation development

**Figure 2:**
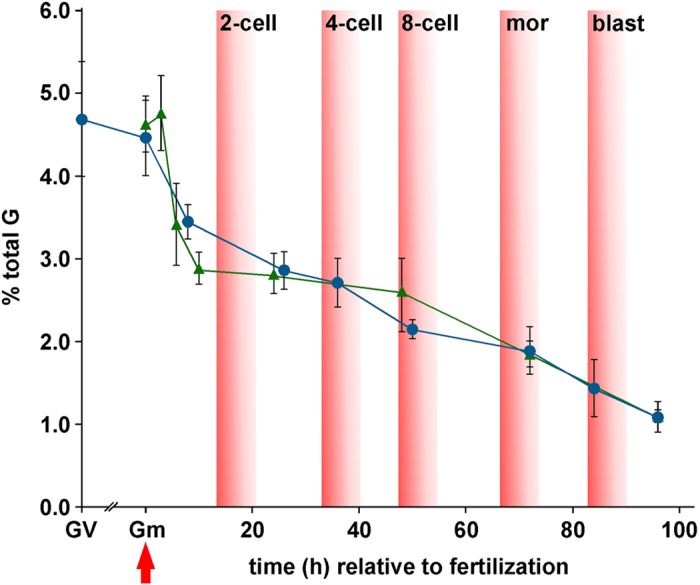
Quantification by SMM of 5 mC during mouse preimplantation development. Germinal vesicle (GV) oocyte and preimplantation embryo 5 mC content (Y-axis) are plotted against the approximate time (h) after fertilization (red arrow). Cell numbers in early (~84 h) and late (~96 h) blastocysts were 32.4 ± 4.23 (*n* = 12) and 63.7 ± 13.0 (*n* = 11) respectively. Values are for embryos produced by natural mating (blue circles) and IVF (green triangles). Zero hour values are shown for gametes (Gm) either for mII oocytes (blue circle) or an average of sperm plus mII oocyte (green triangle). Approximate times at which different developmental stages appear are also indicated. mor, morula; blast, blastocyst. Mean values are ±sd (3 ≤ *n* ≤ 15). Embryo numbers per analysis are given in Supplementary Table S1.

Quantification by SMM of 5 mC in immature, germinal vesicle (GV, prophase I stage) oocytes revealed that levels were similar to those of mII oocytes (4.69 ± 0.70% *vs* 4.46 ± 0.46%; *p* = 0.463) (Fig. 2; Supplementary Table S1; see also below). Genomic DNA from caput epididymidal tissue contained a lower 5 mC content than that of mature, cauda epididymidal sperm used in subsequent *in vitro* experiments (caput tissue *vs* cauda sperm, 4.03 ± 0.08 *vs* 4.80 ± 0.22%, *p* < 0.0001; Supplementary Fig. S1c and Supplementary Table S1).

We applied SMM to embryos produced by natural mating in a time-course, from fertilization to blastocyst development at embryonic day 4.5 (E4.5) (3 ≤ *n* ≤ 5). 5 mC levels decreased to 3.45 ± 0.21% after 6 ~ 8 h (1-cell embryos) and more gradually thereafter to 1.09 ± 0.19% by ~96 h (Fig. 2).

Because embryo culture has been reported to affect DNA methylation^38^, we determined 5 mC profiles during preimplantation development following *in vitro* fertilization (IVF). We also evaluated whether the first polar body (Pb_1_) contributed to 5 mC levels. Irrespective of whether a Pb_1_ was associated with the oocyte at the time of fertilization, 5 mC level profiles in embryos following IVF and natural mating were superimposable (Fig. 2). For example, values were similar after ~24 h (*p* = 0.823) and 48 ~ 50 h (*p* = 0.070) (Fig. 2). Levels of 5 mC had dropped substantially to 2.89 ± 0.20% after 10 h (*p* < 0.0001 *vs* mII oocytes; *n* = 6) (Fig. 2), suggesting that a major phase of 5 mC removal occurred prior to and during mitotic S-phase, which initiates ~8 h post-fertilization^39^, with the first cell division after 14 ~ 15 h. The global genomic decline in 5 mC levels between 6 and 24 h in IVF was only ~17.5% (Fig. 2), a value that is too small to be accounted for entirely by passive demethylation following DNA replication, and little or no decline of 5 mC was observed from 10 to 48 h. Although linear regression analyses of subsequent development best fit a model in which 5 mC decreased with each cycle of DNA replication (*R*^2^ = 0.945; Fig. 2)^40^, the rate of decline accelerated slightly from the 8-cell stage. These patterns seem to reflect a complex balance of processes to maintain global 5 mC homeostasis during 1-cell and cleavage stages, including protection from demethylation (as, for example, occurs at imprinted loci), active and passive demethylation and maintenance methylation (E3.5; Fig. 2)^18^,^21^,^22^. We therefore investigated the relative contributions of these processes to different parental genomes in 1- and 2-cell embryos generated by manipulation.

### Biparental 5 mC removal prior to the first mitotic S-phase

**Figure 3:**
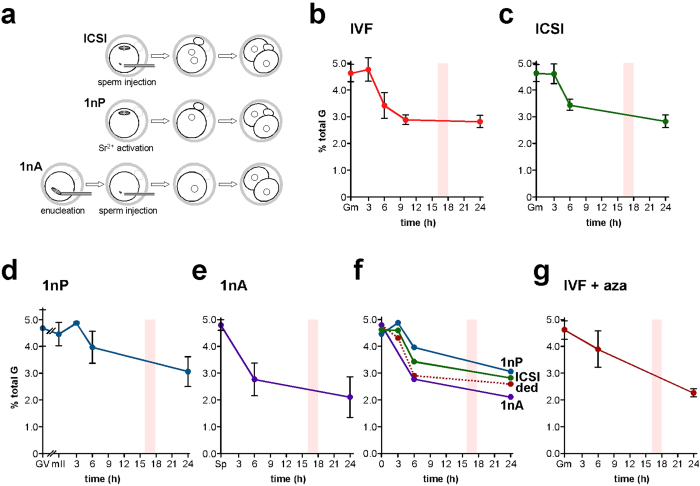
5 mC profiles determined by SMM in mouse embryos produced by *in vitro* fertilization and different types of micromanipulation. (**a**) Schematic representation of three of the principal types of manipulation used to generate embryos: intracytoplasmic sperm injection (ICSI), parthenogenesis (1nP) and androgenesis (1nA). (**b**) Levels of 5 mC determined by SMM and expressed as a percentage of total G-residues in embryos at the times shown (h) after activation of development following *in vitro* fertilization (IVF). (**c**) 5 mC levels as per (**b**) except that embryos were generated by ICSI, (**d**) parthenogenesis (1nP), or (**e**) androgenesis (1nA). (**f**) Composite of panels (**c–e**) showing deduced values (ded) for 5 mC levels in androgenotes. Model 1 (Supplementary Fig. S4) was used to produce the estimated 1nA values but models 1 and 2 gave similar results. mII, metaphase II oocyte; Sp, cauda epididymidal sperm. (**g**) 5 mC levels as per panel (**b**), except that IVF was performed in the presence of the DNA methyl-transferase inhibitor, 5-aza-2′deoxycytidine (azaC). A bar in each histogram marks the approximate time of the first cell division. The zero time-point shows values for mII oocytes in (**d**,**f**) and the average of mII oocytes plus sperm for (**b**,**c**,**f**,**g**) and sperm for (**e**). Mean values are ±sd. Embryo numbers per analysis are given in Supplementary Table S1.

Accurate determination of 5 mC in early embryos requires synchronized cohorts, yet the time of sperm-oocyte union in IVF can only be approximated. One way to achieve synchronization is to inject sperm (intracytoplasmic sperm injection, ICSI) at precisely-indicated times (Fig. 3a). Moreover, although ICSI is the method of choice in human assisted reproduction^41^, there are few analyses of whether ICSI affects DNA reprogramming in mammalian early embryos^42^. We therefore applied SMM to the first 24 h of mouse development after ICSI; in our hands, >70% of ICSI-derived 1-cell embryos develop to term showing that most have full developmental potential. IVF and ICSI 5 mC profiles were similar over the first 24 h (Fig. 3b,c); 5 mC content 3, 6 and 24 h post-ICSI departed by <3.4% from values at corresponding times in IVF. This is consistent with normal genomic DNA methylation kinetics in the first mitotic cell cycle of ICSI-derived embryos.

We next manipulated embryos to determine the kinetics of 5 mC in each parental genome. Although immunofluorescence microscopy previously lead to the view that maternal 5 mC levels remain constant in 1-cell mouse embryos^21^,^23^,^27^, bisulfite sequence analysis has also suggested that the maternal genome undergoes a net loss of 5 mC during this period^18^,^22^. We investigated this dichotomy by quantifying maternal genomic 5 mC in haploid parthenogenotes (*ie* embryos containing only a maternally-derived genome) (Fig. 3d, 1nP). Parthenogenetic 5 mC content diminished from 4.46 ± 0.46% to 3.97 ± 0.62% (11.0%, *p* = 0.073) by 6 h after the initiation of development (activation). This decrease was indicative of active, pre-S-phase 5 mC depletion from the maternal genome. The 5 mC content in GV oocytes was similar to that of mature mII oocytes (4.69 *vs* 4.46%, *p* = 0.463; Fig. 3d). The 5 mC content of 2-cell parthenogenotes 24 h post-activation was also similar to that of stage-matched 2-cell IVF and ICSI embryos, showing that paternal and maternal genomes possessed similar levels of 5 mC by 24 h (*p* = 0.385 *vs* IVF, *p* = 0.572 *vs* ICSI) (Fig. 3b–d).

We obtained estimates of paternal 5 mC levels in two ways. First, we measured 5 mC by SMM in androgenotes produced by injecting sperm into enucleated mII oocytes (Fig. 3e). Secondly, we estimated paternal 5 mC content indirectly by harnessing data from 1-cell embryos and parthenogenotes to produce a subtractive zygotic paternal 5 mC profile. The profiles generated by the two methods were in good accordance, with slightly higher deduced values (Fig. 3f), suggesting that the absence of the maternal genome in androgenotes (1nA) did not greatly impact 5 mC levels in the paternal genome (Fig. 3f). Taking values that were either deduced or determined directly from androgenotes (Fig. 3e), paternal 5 mC content declined a little from that in cauda epididymidal sperm for at least 3 h post-fertilization (4.80% *vs* deduced 4.32%) but then underwent a rapid decline from 4.80 ± 0.22% to an experimental value of 2.77 ± 0.63%, a drop of ~42% (*p* = 0.0005) 3 ~ 6 h post-injection (Fig. 3f). Paternal 5 mC content changed very little from 6 ~ 24 h, a period straddling the first mitotic division (*p* = 0.286; Fig. 3e), suggesting that 5 mC methylation compensates for passive demethylation during the first S-phase and 1- to 2-cell division.

### Simultaneous post-meiotic paternal genome methylation and demethylation

To investigate paternal DNA methylation in the first embryonic cell cycle, we performed IVF in the presence of the DNA methyl-transferase (Dnmt) inhibitor, 5-aza-2′deoxycytidine (azaC). 5 mC levels 6 h post-IVF were similar in the presence or absence of azaC (*p* = 0.246; Fig. 3b,g), suggesting that little or no net DNA methylation normally occurs from 0 to 6 h. However, by 24 h, 5 mC levels in the azaC group were 80.5% of controls (*p* = 0.005; Fig. 3b,g). This suggests that the decline in 5 mC 6 ~ 24 h post-fertilization is accompanied by balancing *de novo* or maintenance DNA methylation.

### Early embryonic genome 5 hmC profiles

**Figure 4:**
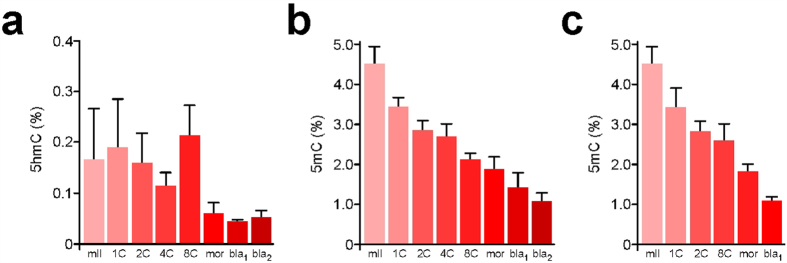
Quantification by SMM of 5 hmC during mouse preimplantation development. (**a**) Content (% total G) of 5 hmC (Y-axis) in metaphase II oocytes (mII) and preimplantation embryos at different preimplantation developmental time points. bla_1_ and bla_2_ respectively refer to blastocysts ~84 h and ~96 h post-fertilization. Mean values are ±sd (3 ≤ *n* ≤ 5). Embryos were produced by natural mating. 5 mC levels in embryos produced by natural mating (**b**) or IVF (**c**) are shown for comparison, with mean values shown ±sd (3 ≤ *n* ≤ 7). Embryo numbers are given in Supplementary Table S1.

**Figure 5:**
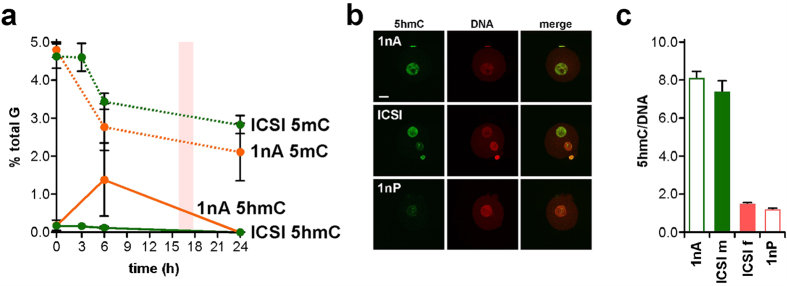
Comparative 5 mC and 5 hmC profiles for ICSI and androgenesis. (**a**) Levels of 5 mC (dotted lines) and 5 hmC (solid lines) determined by SMM and expressed as a percentage of total G-residues in embryos at the times shown (h) after activation. A value of 0.00 was taken for samples in which 5 hmC was not detected. Embryos were generated by intracytoplasmic sperm injection (ICSI, green) or androgenesis (1nA, orange). Data (2 ≤ *n* ≤ 3) are expressed as means (±sd). Zero hour time points show the average of combined values for sperm plus mII oocyte (ICSI) or sperm alone (1nA). (**b**) Fluorescence images of a haploid androgenote (1nA, top row), an embryo generated by ICSI (central row) and a haploid parthenogenote (1nA) 6 h after sperm injection (1nP and ICSI) or parthenogenic activation (1nP). Embryos were labeled with anti-5 hmC antibodies (green) and propidium iodide (DNA, red). Scale bar, 10 μm. (**c**) Pixel quantification (± s.e.m.) of *n* = 5 samples of (**b**), showing fluorescence levels normalized against PI (DNA). For paternal pronuclei in 1nA and ICSI (ICSI m), and maternal pronuclei in 1nP and ICSI (ICSI f), *p* > 0.05. Embryo numbers per analysis are given in Supplementary Table S1.

Quantification by SMM can reveal relationships between 5 mC and 5 hmC content in early development. Levels of 5 hmC were 0.18 ± 0.16% of total G (*n* = 3) in sperm and 0.15 ± 0.09% (*n* = 6) in mII oocytes (Fig. 4a). Following natural mating, steady-state 5 hmC levels progressively declined from 0.19 ± 0.09% in 1-cell embryos (*n* = 3) to 0.05 ± 0.01% in late blastocysts (*n* = 5) with an unexplained increase (*p* = 0.036, *n* = 4) from 4- to 8-cell stages (Fig. 4a). The 1-cell 5 hmC content was similar to 6 h values in IVF (0.12%, *n* = 2) and ICSI (0.12 ± 0.05%, *n* = 5), suggesting that over-all, 5 hmC does not accumulate in 1-cell embryos in the first 6 h^27^,^29^,^43^. 5 hmC can be oxidatively converted by Tet enzymes into 5-formylC (5fC) and 5-carboxylC (5caC)^34^, but we were unable to detect 5fC or 5caC ~8 h post-fertilization, with respective quantification thresholds of 480 amol and 360 amol (Supplementary Fig. S3). Our measurements suggest that average parental 5 hmC content was 0.16% (but *n* = 1 in which 5 hmC was detected) 3 h post-ICSI and 0.12 ± 0.05% (*n* = 5) after 6 h (Fig. 5a). These values are approaching detection threshold levels and should be interpreted with caution. However, our measurements provided an average that did not distinguish between levels in each parental genome. To address this directly, we determined 5 hmC levels in androgenotes. We detected 5 hmC levels of 1.35% in androgenotes 6 h post-ICSI, reflecting a ~9-fold increase (*n* = 3; Fig. 5a). This marked increase in the amount of genomic 5 hmC in androgenotes but not in biparental embryos was too great to be accounted for by a depletion of similar magnitude in the maternal genome. Immunofluorescence microscopy (Fig. 5b,c) suggested that 5 hmC was abundant in androgenotes and the male pronuclei of ICSI embryos, but produced a net over-estimate of 5 hmC in ICSI embryos compared to SMM that warrants further investigation (Fig. 5c).

## Discussion

We here employed SMM to quantify maternal and paternal 5 mC and 5 hmC content prior to and immediately after the activation of development, revealing definitive epigenetic dynamics in early mouse embryos. Such comparative profiling is problematic with immunofluorescence microscopy, which is affected by chromatin states, particularly compaction in sperm and mII oocytes. For example, 5 mC levels in mII oocytes and immediately post-fertilization have not been compared^23^. Moreover, levels of 5 mC and 5 hmC determined by quantitative immunofluorescence using different antibodies cannot be compared. By contrast, SMM enables the simple, direct measurement of absolute 5 mC and 5 hmC levels that may be compared in a given sample.

This addition of more detailed measurements addresses prevailing models of early embryonic epigenetic regulation and delineates conditions that they must satisfy. Both bisulfite sequencing and immunofluorescence microscopy indicate a drop in zygotic genome methylation prior to the first mitotic S-phase^15^,^17^,^23^. The extent of demethylation is at variance depending on the conventional method used, with bisulfite sequencing typically suggesting a smaller decrease^23^,^29^ (discussed in ref. 44). This might *a priori* be because bisulfite chemistry in earlier work has not always distinguished between 5 mC, 5 hmC or other modifications^45^ but SMM suggests that the contribution from 5 hmC is extremely small: typically ~0.2%. Although the precise values at these near-threshold levels should be interpreted with caution, this illustrates the principle that SMM is applicable to multiple DNA modifications in a given sample, unlike either bisulfite sequencing or immunofluorescence microscopy, which requires a predetermined and discriminatory antibody.

SMM lacks the intracellular resolution required for parental genome discrimination, but this limitation could be addressed by analyzing parthenogenotes or androgenotes, which respectively contain only maternal and paternal genomes (Fig. 3d–f). The results confirm active paternal and maternal 5 mC depletion prior to mitotic S-phase; active depletion of maternal 5 mC was inferred recently from bisulfite sequence analyses^18^,^22^. In one reciprocal model^23^, zygotic 5 mC is removed by oxidation to 5 hmC: paternal genomic 5 mC content declines as 5 hmC content increases (*ie* producing reciprocity) and in Tet3-deficient zygotes, paternal 5 hmC levels are low and 5 mC levels high^19^,^23^. Profiles of 5 mC and 5 hmC produced by SMM may exhibit this reciprocal relationship over all in normal biparental 1-cell embryos (Fig. 5), but do not distinguish between parental genomes. However, our quantification sets an upper limit on the level of paternal 5 hmC increase of ~0.3% in normal 1-cell embryos (Fig. 5). A 5 hmC level increase of this magnitude (0.3%) would be smaller than the observed decrease in 5 mC in ICSI (4.60 ± 0.39 *vs* 3.44 ± 0.23%, 3 *vs* 6 h; Fig. 3c), suggesting either anomalously rapid dynamics of 5 hmC removal^43^ or that there is no strong mechanistic correlation between 5 mC and 5 hmC from 3 to 6 h post-fertilization. SMM is thus consistent with previous immunofluorescence studies suggesting that demethylation precedes S-phase and occurs independently of hydroxymethylation and pronucleus formation^17^,^46^.

A straightforward reciprocal relationship between 5 mC and 5 hmC is also challenged by high-resolution spatiotemporal immunofluorescence microscopy of zygotic 5 hmC and 5 mC^27^,^47^. Enforced Tet3 expression *in vivo* causes an increase in 5 hmC with no obvious decrease in 5 mC^48^. Furthermore, we have shown that maternal 5 mC content declines 3 ~ 6 h post-activation of development (Fig. 3d) even though the maternal pronucleus lacks Tet3 (ref. 19)suggesting that the maternal genome undergoes Tet3-independent 5 mC depletion, possibly involving Tet1 (ref. 48). Collectively, our findings suggest that 5 hmC is not necessarily an oxidative intermediate in zygotic 5 mC removal, and 5 hmC may constitute a discrete gene-regulatory mark associated with 5 hmC-binding proteins with roles in early development^49^,^50^. However, we noted a marked increase in 5 hmC levels in androgenotes coincidental with the decline in 5 mC levels (Fig. 5a). There is no evidence for or against a causal relationship between 5 mC and 5 hmC level changes in androgenotes in these data, but they point to a *trans*-regulatory role of paternal 5 hmC levels by the maternal genome or its associated (*eg* pronuclear) components. In sum, the reciprocal model of 5 mC oxidation to 5 hmC by Tet3 does not explain the levels or distributions of each.

We have demonstrated the utility of SMM for 5 mC, 5 hmC, 5fC and 5caC, but expect that the method can be extended to any poly-nucleic acid modification or adduct, including *N*^6^-methyladenosine in RNA, *N*^6^-methyladenine and 4 mC in prokaryotic DNA and mutagens that include so-called DMBA-DNA and etheno-DNA adducts. The analytical power of SMM in small samples promises to facilitate monitoring of anti-cancer chemotherapy based on DNA cross-linking agents such as Cisplatin^51^,^52^. The association of global DNA hypomethylation with pluripotency^37^ suggests that SMM could provide a rapid quantitative cellular potency indicator in small population samples. Locus-specific SMM should also be possible following chromatin immunoprecipitation (ChIP) with antibodies against chromatin binding proteins: SMM-ChIP.

## Methods

### Chemicals

2′-Deoxyguanosine (G) was obtained from Sigma-Aldrich Co. (St. Louis, MO). ^15^N_5_-2′-Deoxyguanosine (^15^N_5_-G) was purchased from Cambridge Isotope Laboratories Inc. (Andover, MA). 5-Methyl-2′-deoxycytidine (5 mC) was obtained from Tokyo Chemical Industry Co., Ltd. (Tokyo, Japan). 5-(Methyl-*d*_*3*_)-2′-deoxycytidine (5 mC-*d*_*3*_) was purchased from Toronto Research Chemicals Inc. (Ontario, Canada). 5-Hydroxymethyl-2′-deoxycytidine (5 hmC), 5-formyl-2′-deoxycytidine (5fC) and 5-carboxyl-2′-deoxycytidine (5caC) were from Berry and Associates Inc. (Dexter, MI). 5-(Hydroxymethyl-*d*_*2*_)-2′-deoxycitidine (5 hmC-*d*_*2*_) was synthesized at Meijo University. All other chemicals used were of the highest grade commercially available.

### ES cell culture

Mouse embryonic stem (ES) cells ES-D3 obtained from ATCC were cultured on mitomycin C-treated mouse embryonic fibroblast. Medium was consisted with knockout Dullbecco’s modified Eagle medium (Gibco) containing 15% knockout serum replacement (KSR, Gibco), 1% antibiotics, 1% non-essential amino acids, 2 mM glutamine, 0.1 mM 2-mercaptoethanol, and 1000 U/ml leukemia inhibitory factor (LIF, Wako). Culture medium was changed every day and cells were sub-cultured every 3–4 d.

### Experimental animals

All animal experiments strictly observed national statutes and local institutional guidelines. Mice (B6D2F_1_ and ICR) were supplied by SLC (Shimizu Laboratory Supplier, Kyoto) or CLEA (Tokyo) in Japan or were bred from stocks in-house or otherwise supplied by Charles River (L’Arbresle, France) (UK). Tissues from cynomolgus monkeys (*Macaca fascicularis*) were obtained from the Tsukuba Primate Research Center (TPRC), National Institutes of Biomedical Innovation (NIBIO) according to approval from, and guidelines of, the NBIO Animal Care Committee. Mouse maintenance and usage was according to the law and in observance of host institution guidelines.

### Preparation and culture oocytes and embryos

Eight-to-10-week-old B6D2F_1_ females were superovulated using standard serial injections of pregnant mare serum gonadotropin (PMSG) followed 48 h later by human chorionic gonadotropin (hCG). 14 ~ 16 h after hCG injection, oviductal metaphase II (mII) oocytes were collected in M2 medium and cumulus cells removed by hyaluronidase (Sigma) treatment as described previously^17^,^53^. Randomly-selected mII oocytes and mII oocytes with no extant first polar body (Pb_1_) gave similar values for 5 mC. For 96 h profiles, embryos were obtained by *in vitro* fertilization (IVF, see below) or by naturally mating superovulated B6D2F_1_ females and B6D2F_1_ males. For IVF, freshly-isolated cauda epididymidal sperm were incubated in 100 μl pre-warmed human tubal fluid (HTF, Millipore) for 1 h and following sperm dispersal, 1 ~ 3 μl of the suspension transferred to 200 μl of HTF containing cumulus oophorous complexes. After 2 h, nascent embryos were washed thoroughly and cultured in KSOM (Millipore) supplemented with 1 mg/ml BSA (Sigma). For natural mating, 2-cell embryos were collected into M2 medium from plugged females 1.5 days *post coitum* (dpc) and transferred to pre-warmed KSOM (Millipore) containing 1 mg/ml BSA for continued culture. In all experiments, oocyte incubation and embryo culture were in humidified CO_2_ [5% (v/v) in air] at 37 °C. Prior to analysis, samples were collected in a minimum volume and stored at −80 °C in 1.5 ml tubes.

### Genomic DNA extraction and treatment

Genomic DNA extraction from tissues and cultured cells employed a spin column (high pure PCR template preparation kit, Roche Mannheim, Germany) according to the instruction manual. Cauda epididymidal sperm from of 8–12 week old B6D2F_1_ males were washed once in PBS prior to DNA extraction. DNA from gametes, embryos and in experiments using 10 to 1000 ES-D3 cells was extracted on ultra-microspin columns (nucleospin tissue XS, Macherey-Nagel GmbH and Co. KG, Duren, Germany) and the total recovered DNA (4.08 ± 0.66 ng from 1000 cells) subjected to small-scale lc-ms/ms (SMM). Genomic DNA digestion was performed as reported with minor modifications^54^. Briefly, <1 μg DNA was digested at 37 °C for 3 h with nuclease P1 (4U, Wako Pure Chemical Industries, Ltd., Osaka, Japan) and alkaline phosphatase (3U, Wako) in 100 μl of buffer mixture (3 mM sodium acetate [pH 5.3] containing 1 mM 2-mercaptoethanol, 2 mM ZnSO_4_) followed by the addition of 20 μl 50 mM Tris-HCl (pH8.5) and continued incubation for 3 h. Enzymes were methanol-precipitated, and the supernatant containing nucleoside evaporated and stored at −80 °C. Each digest was reconstituted in 100 μl of internal standard solution containing 5 mC-*d*_*3*_ (0.1 nM), 5 hmC-*d*_*2*_ (0.1 nM) and ^15^N_5_-G (1 nM) before being subjected to SMM.

### DNA analysis by SMM

Analysis of genomic DNA samples by small-scale ms/ms (SMM) was performed on high performance liquid chromatography (HPLC) (Prominence series, Shimadzu, Kyoto, Japan) equipped with a triple quadrupole mass spectrometer (API4000 system, AB Sciex, Foster City, CA). Sample aliquots (60 μl) were separated at 40 °C using a reverse-phase column (TSKgel ODS-100V, 4.6 mm × 75 mm × 3 μm; Tosoh, Tokyo, Japan) in isocratic mode with a mobile phase (methanol-10 mM ammonium formate [20:80 {v/v}]) at a flow rate of 0.3 ml/min. Where target-ionization was insufficient, gradient HPLC elution (mobile phase A, 10 mM ammonium formate; mobile phase B, methanol; gradient mode, 0–12 min, linear gradient to 15% B; 12–15 min, isocratic with 15% B; 15–20 min, linear gradient to 30% B) was sometimes performed to remove impurities and residual buffer from the sample.

SMM is adapted for ultrasensitive quantification of 5 mC and 5 hmC in three principal ways. First, the triple-quadropole mass spectrometer is specialized for high-sensitivity quantification. Secondly, the buffer concentration employed for enzymatic digestion of DNA was reduced 10-fold relative to standard protocols, allowing efficient ionization of target molecules. Finally, HPLC was optimized by employing a short column (75 mm × 4.6 mm inner diameter) packed with small particles (3 mm diameter). This avoided diffusion of target compounds and permitted isocratic elution for rapid and simultaneous analysis of 5 mC and 5 hmC. Stable, isotope-labeled nucleosides were used as internal standards for 5 mC, 5 hmC and G.

Elution of 5 mC, 5 hmC and G was respectively at 4.1, 5.0 and 5.3 min on isocratic mode (total running time, 12 min) or respectively at 9.8, 13.3 and 14.4 min on gradient mode (20 min). Mass spectral analysis was carried out in positive ion mode with nitrogen as the nebulizing gas. Ionization was performed under the following conditions: curtain gas, 10; collision gas, 8; ion source gas 1, 60; ion source gas 2, 60; ion source voltage, 4500 V; ion source temperature, 400 °C. Positive ions were acquired in MRM mode. MRM transitions were monitored as follows: 5 mC (*m/z* 242 → 126), 5 mC-*d*_*3*_ (*m/z* 245 → 129), 5 hmC (*m/z* 258 → 142), 5 hmC-*d*_*2*_ (*m/z* 260 → 144), G (*m/z* 268 → 152), ^15^N_5_-G (*m/z* 273 → 157). The proportion (%) of C that was 5 mC or 5 hmC in each sample was given by: % 5 mC or 5 hmC  =  [(value for 5 mC or 5 hmC)/(value for G)]x 100. The mouse genome contains 21% C^55^.

In some cases, 5 hmC content was measured using a Xevo TQ-S (Waters, Manchester, UK) with an ACQUITY UPLC system (Waters). Sample aliquots (10 μl) were separated at 40 °C on a CORTECS HILIC column 2.1 mm × 50 mm, 1.7 μm (Waters) at a flow rate of 0.5 ml/min and subsequently eluted as follows (solvent A, 10 mM ammonium acetate; solvent B, acetonitrile): 0–1.7 min, isocratic with 95% B; 1.7–4.5 min, linear gradient to 20% B. MRM was performed in positive ion mode using nitrogen as the nebulizing gas. Experimental conditions were as follows: ion source temperature, 150 °C; desolvation temperature, 650 °C; desolvation gas flow rate, 1000 L/h; capillary voltage, 3.0 kV; cone voltage, 10 V; cone gas flow rate, 150 L/h; collision gas, argon; collision gas flow rate, 0.12 ml/min.

All SMM analysis was performed within 3 months of genomic DNA sample preparation.

### Statistical analysis

Statistical analysis was performed to evaluate significance between two groups using Chi-squared or Student’s *t*-tests. A two-tailed *p* < 0.05 was regarded as statistically significant.

## Additional Information

**How to cite this article**: Okamoto, Y. *et al.* DNA methylation dynamics in mouse preimplantation embryos revealed by mass spectrometry. *Sci. Rep.* **6**, 19134; doi: 10.1038/srep19134 (2016).

## Supplementary Material

Supplementary Information
