# Supplementary material for: DNA methylation dynamics in mouse preimplantation embryos revealed by mass spectrometry
Source: Sci Rep. 2016 Jan 11;6:19134. doi: 10.1038/srep19134 (PMC4707515; doi:10.1038/srep19134)
Supplement: Supplementary Information [file srep19134-s1.pdf]

Supplementary figures and table for

DNA methylation dynamics in mouse preimplantation embryos revealed by mass spectrometry

Yoshinori Okamoto[1], Naoko Yoshida[2], Toru Suzuki[3], Nobuhiro Shimozawa[4], Maki Asami[3], Tomonari Matsuda[5], Nakao Kojima[1], Anthony C. F. Perry[3†], and Tatsuyuki Takada[6†]

[†]Correspondence and requests for materials should be addressed to A.C.F.P. (perry135@aol.com) or T.T. (ttakada@ph.ritsumei.ac.jp).

This PDF file includes

Figures S1 to S4

Figure Legends S1 to S4

Table S1

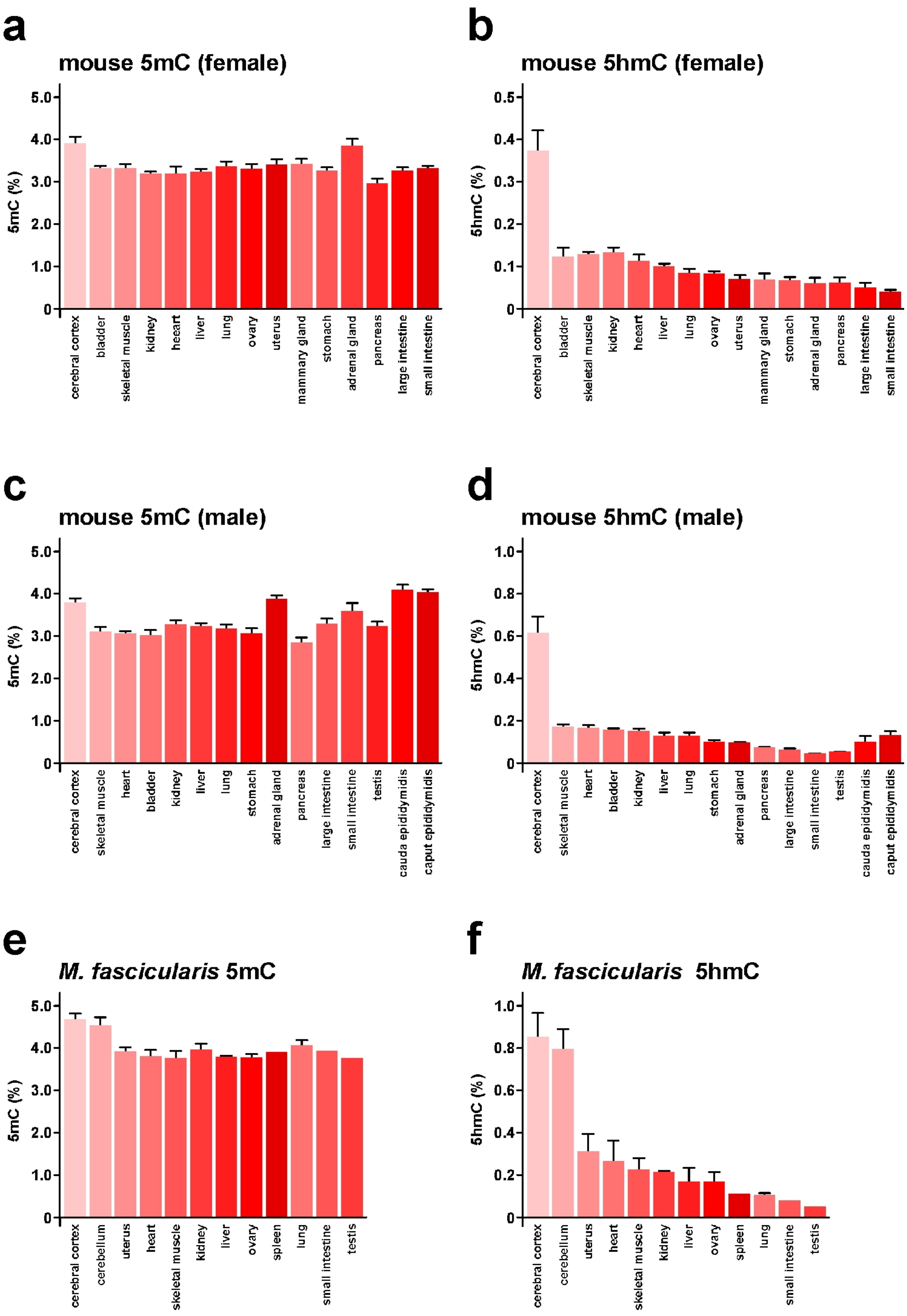
a
mouse 5mC (female)
5mC (%)
0
1.0
2.0
3.0
4.0
5.0
cerebral cortex
bladder
skeletal muscle
kidney
heeart
liver
lung
ovary
uterus
mammary gland
stomach
adrenal gland
pancreas
large intestine
small intestine
b
mouse 5hmC (female)
5hmC (%)
0
0.1
0.2
0.3
0.4
0.5
cerebral cortex
bladder
skeletal muscle
kidney
heeart
liver
lung
ovary
uterus
mammary gland
stomach
adrenal gland
pancreas
large intestine
small intestine
c
mouse 5mC (male)
5mC (%)
0
1.0
2.0
3.0
4.0
5.0
cerebral cortex
skeletal muscle
heart
bladder
kidney
liver
lung
stomach
adrenal gland
pancreas
large intestine
small intestine
testis
cauda epididymidis
caput epididymidis
d
mouse 5hmC (male)
5hmC (%)
0
0.2
0.4
0.6
0.8
1.0
cerebral cortex
skeletal muscle
heart
bladder
kidney
liver
lung
stomach
adrenal gland
pancreas
large intestine
small intestine
testis
cauda epididymidis
caput epididymidis
e
M. fascicularis 5mC
5mC (%)
0
1.0
2.0
3.0
4.0
5.0
cerebral cortex
cerebellum
uterus
heart
skeletal muscle
kidney
liver
ovary
spleen
lung
small intestine
testis
f
M. fascicularis 5hmC
5hmC (%)
0
0.2
0.4
0.6
0.8
1.0
cerebral cortex
cerebellum
uterus
heart
skeletal muscle
kidney
liver
ovary
spleen
lung
small intestine
testis

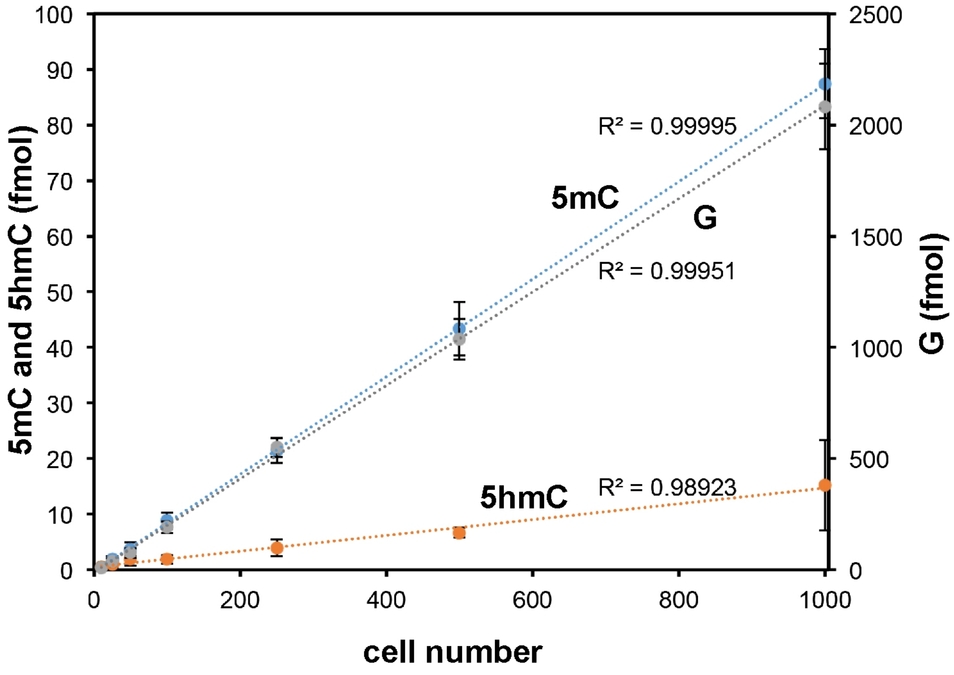
100
90
80
70
60
50
40
30
20
10
0
5mC and 5hmC (fmol)
2500
2000
1500
1000
500
0
G (fmol)
0
200
400
600
800
1000
cell number
R² = 0.99995
5mC
G
R² = 0.99951
5hmC
R² = 0.98923

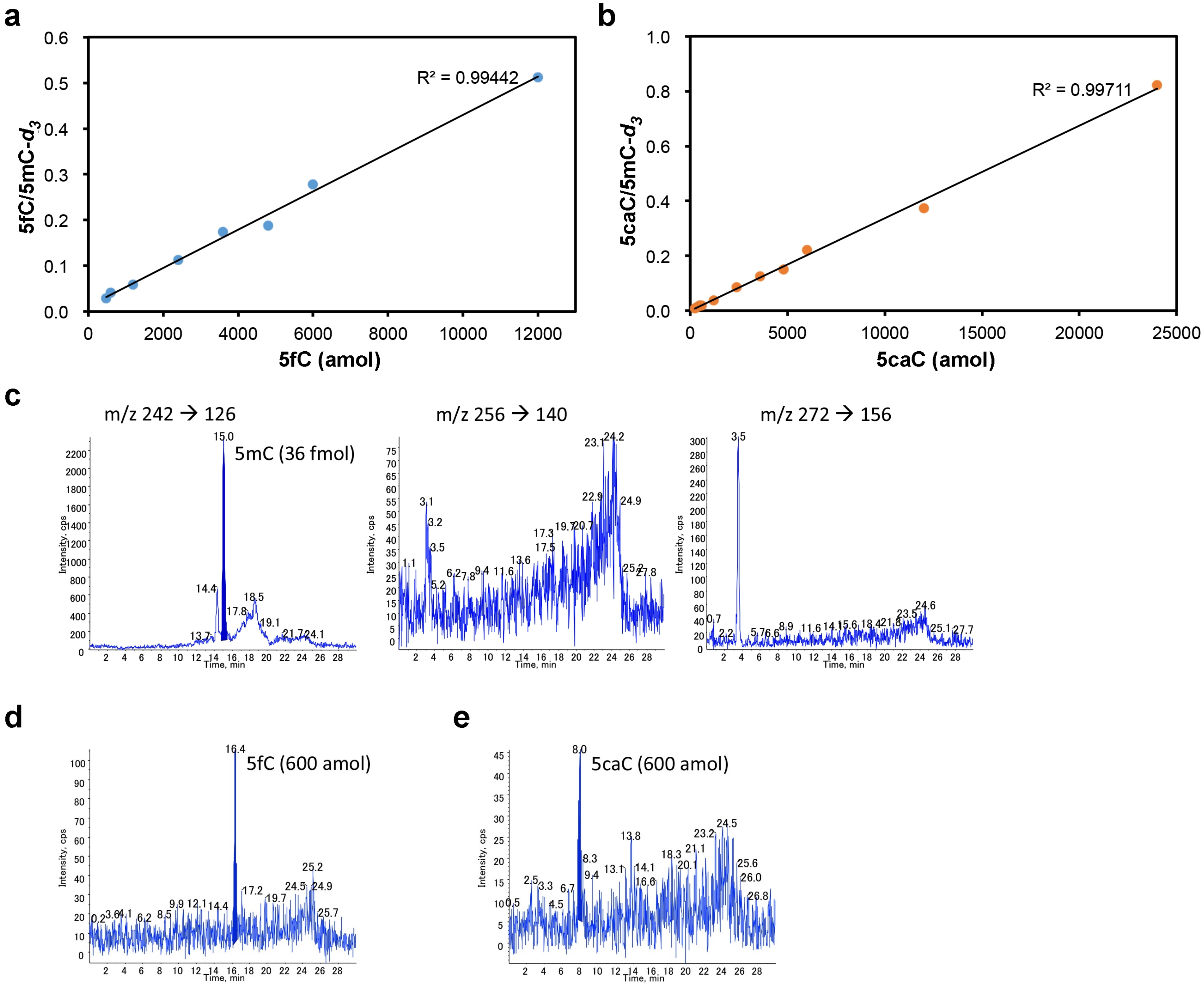

a
R² = 0.99442
5fC/5mC-d3
5fC (amol)
b
R² = 0.99711
5caC/5mC-d3
5caC (amol)
c
m/z 242 → 126
5mC (36 fmol)
m/z 256 → 140
m/z 272 → 156
Intensity, cps
Time, min
d
5fC (600 amol)
e
5caC (600 amol)

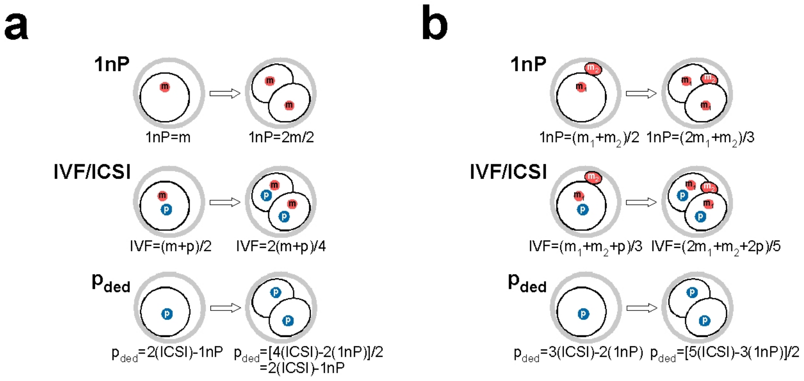
a
1nP
1nP=m
1nP=2m/2
IVF/ICSI
IVF=(m+p)/2
IVF=2(m+p)/4
pded
pded=2(ICSI)-1nP
pded=[4(ICSI)-2(1nP)]/2
=2(ICSI)-1nP
b
1nP
1nP=(m1+m2)/2
1nP=(2m1+m2)/3
IVF/ICSI
IVF=(m1+m2+p)/3
IVF=(2m1+m2+2p)/5
pded
pded=3(ICSI)-2(1nP)
pded=[5(ICSI)-3(1nP)]/2

Supplementary Table S1 Values of 5-methyl-2'-deoxycytidine (5mC) and 5-hydroxymethyl-2'-deoxycytidine (5hmC)

Sample	time (h) (stage)*	5mC mean	5mC sd	5hmC mean	5hmC sd	Samples, *n*	embryos/sample (*n*)
sperm	-	4.80	0.22	0.18	0.16	4	-
mII[¶]	-	4.46	0.46	0.15	0.09	15	137, 110, 100, 101, 100, 123, 132, 132, 92, 120, 108, 100, 100, 100, 106
IVF	3	4.76	0.46	nd	-	3	100, 100, 106
	6	3.42	0.50	0.12	-	7	100, 100, 93, 104, 110, 107, 106
	10	2.89	0.20	0.07	0.02	6	100, 100, 100, 101, 121, 146
	24	2.82	0.25	nd	-	6	100, 100, 56, 74, 50, 42
	48	2.61	0.40	nd	-	4	16, 16, 15, 16
	72	1.85	0.16	nd	-	3	10, 10, 10
	96	1.10	0.08	nd	-	4	7, 7, 7, 8
IVF+azaC	6	3.90	0.70	0.45	-	3	100, 100, 100
	24	2.27	0.17	0.10	-	3	50, 50, 50
	48	2.97	0.64	0.82	-	1	38
1nP	3	4.88	0.05	0.26	-	3	100, 100, 100
	6	3.97	0.62	nd	-	5	100, 100, 100, 100, 120
	24	3.06	0.58	nd	-	4	100, 100, 50, 100
1nP+azaC	6	3.62	0.25	nd	-	1	128
1nA	6	2.77	0.63	1.38	0.98	3	100, 100, 100
	24	2.11	0.78	nd	-	2	100, 100
ICSI	3	4.60	0.39	0.16	-	3	100, 100, 100
	6	3.44	0.23	0.12	0.05	5	100, 100, 100, 100, 150
	24	2.83	0.26	nd	-	3	100, 100, 100
natural	8 (1C)	3.45	0.21	0.19	0.09	3	235, 213, 150
mating	26 (2C)	2.86	0.23	0.16	0.06	3	130, 113, 93
	36 (4C)	2.71	0.30	0.11	0.03	3	50, 50, 50
	50 (8C)	2.15	0.12	0.22	0.06	4	30, 28, 28, 22
	72 (mor)	1.89	0.29	0.06	0.02	3	142, 23, 93
	84 (B)	1.44	0.35	0.05	0.00	4	38, 15, 15, 70
	98 (eB)	1.09	0.19	0.05	0.01	5	12, 25, 15, 151, 157
GV	-	4.69	0.70	nd	-	3	127, 121, 139
caput	-	4.03	0.08	0.21	0.00	5	-
cauda	-	4.08	0.15	0.19	0.01	5	-

Where $n \leq 2$, replicates are technical replicates. Sperm were from cauda epididymidis.
*Estimated time in hours (h) after activation of development or sperm injection.
¶Abbreviations: mII, metaphase II oocyte; IVF, *in vitro* fertilisation; nd, below detection threshold; azaC, 5-aza-2'deoxycytidine; 1nP, haploid parthenogenetic embryo; 1nA, haploid androgenetic embryo; ICSI, intracytoplasmic sperm injection; 1C-8C, 1- to 8-cell embryo; mor, morula; B, blastocyst; eB, expanded blastocyst; GV, germinal vesicle oocyte; caput, whole caput epididymidal tissue; cauda, whole cauda epididymidal tissue;
